# Characteristics and Influencing Factors of Polycyclic Aromatic Hydrocarbons Emitted from Open Burning and Stove Burning of Biomass: A Brief Review

**DOI:** 10.3390/ijerph19073944

**Published:** 2022-03-25

**Authors:** Hao Zhang, Xuan Zhang, Yan Wang, Pengchu Bai, Kazuichi Hayakawa, Lulu Zhang, Ning Tang

**Affiliations:** 1 Graduate School of Medical Sciences, Kanazawa University, Kakuma-Machi, Kanazawa 920-1192, Japan; zhanghao@stu.kanazawa-u.ac.jp (H.Z.); zhangxuan@stu.kanazawa-u.ac.jp (X.Z.); wangyan@stu.kanazawa-u.ac.jp (Y.W.); baipengchu@stu.kanazawa-u.ac.jp (P.B.); 2 Institute of Nature and Environmental Technology, Kanazawa University, Kakuma-Machi, Kanazawa 920-1192, Japan; hayakawa@p.kanazawa-u.ac.jp; 3 Institute of Medical, Pharmaceutical and Health Sciences, Kanazawa University, Kakuma-Machi, Kanazawa 920-1192, Japan

**Keywords:** biomass composition, burning conditions, emission characteristic, formation mechanism, stove design

## Abstract

To mitigate global warming and achieve carbon neutrality, biomass has become a widely used carbon-neutral energy source due to its low cost and easy availability. However, the incomplete combustion of biomass can produce polycyclic aromatic hydrocarbons (PAHs), which are harmful to human health. Moreover, increasing numbers of wildfires in many regions caused by global warming have greatly increased the emissions of PAHs from biomass burning. To effectively mitigate PAH pollution and health risks associated with biomass usage, the concentrations, compositions and influencing factors of PAH emissions from biomass burning are summarized in this review. High PAH emissions from open burning and stove burning are found, and two- to four-ring PAHs account for a higher proportion than five- and six-ring PAHs. Based on the mechanism of biomass burning, biomass with higher volatile matter, cellulose, lignin, potassium salts and moisture produces more PAHs. Moreover, burning biomass in stoves at a high temperature or with an insufficient oxygen supply can increase PAH emissions. Therefore, the formation and emission of PAHs can be reduced by pelletizing, briquetting or carbonizing biomass to increase its density and burning efficiency. This review contributes to a comprehensive understanding of PAH pollution from biomass burning, providing prospective insight for preventing air pollution and health hazards associated with carbon neutrality.

## 1. Introduction

Polycyclic aromatic hydrocarbons (PAHs) are a group of organic compounds comprising two or more aromatic benzene rings; PAHs are ubiquitous in the atmosphere and have received much attention due to their toxicity to humans [1,2,3]. PAHs entering the human body will be metabolized by cytochrome P450 enzymes and transformed to their quinoid, epoxide and hydroxyl derivatives. Some of the metabolized derivatives show harmful biological activities that are carcinogenic and mutagenic [4,5]. Therefore, exposure to PAHs in the short or long term might cause acute and chronic harmful health effects, including asthma, bronchitis or even lung cancer [2,6].

PAHs are formed through incomplete combustion or pyrolysis of organic materials, such as fossil fuels and biomass [7,8,9]. Fossil fuels are the most widely used energy source, but their burning releases large amounts of greenhouse gases, causing global warming [10,11,12]. With population growth and urbanization, global energy demand will increase by 1.3% every year to 2040 under the current energy policy, which will exacerbate global climate change [13,14]. These rigorous energy issues and the resulting environmental pollution have impelled the transformation of the world energy structure [15,16,17]. Renewable energies (e.g., biofuels, hydroelectricity and nuclear) are considered to mitigate the greenhouse effect [18]. However, some unfavorable factors have restricted the availability of many renewable energy sources, such as hydroelectricity (which is easily affected by climatic conditions) and nuclear energy (nuclear accidents) [19,20]. Biomass fuels stand out from renewable energies due to their low cost, widespread availability and convenience of use; thus, such fuels are receiving increasing attention [21,22].

Biomass fuels mainly come from agricultural and forestry waste such as crop straw, firewood, and animal dung. Unprocessed biomass fuels are all favored fuels in many rural areas because they can be directly used to generate heat or electricity after they are acquired and can be obtained at almost no cost [23,24,25]. According to a previous report, the number of people in the world using biomass directly for cooking reached approximately 2.4 to 3.3 billion in 2018 [23]. In addition, biomass is the fuel that is most widely used in residential space heating, particularly in European countries [26]. Reports show that biomass fuels are usually burned in rudimentary stoves or heaters with poor burning conditions during daily domestic use, favoring PAHs production [27,28,29]. The report shows at least 40,000 premature deaths in Europe every year contributed by biomass combustion for residential space heating [30]. Therefore, although biomass fuel is a recommended carbon-neutral energy source during the energy transition period, the environmental and health effects caused by PAHs emitted from biomass burning cannot be ignored [31,32,33,34]. Apart from human activities, biomass burning also occurs naturally, such as forest and grassland fires caused by intensified global warming. Based on the report of Shen et al., Table 1 briefly summarized the highest PAHs emission industries around the world [35]. The PAHs from biomass burning represented a high proportion of the total emissions, which might lead to relatively high health risks. To promote the eco-friendly and healthy development of biomass fuels, it is crucial to investigate the characteristics of PAHs emitted from biomass burning and their influencing factors [36,37,38,39]. 

Biomass burning can produce PAHs at levels comparable to those of fossil fuel burning [40,41,42]. As shown in Table 2, the PAH emission factors (EFs) of biomass burning are similar to and sometimes even higher than those of coal burning [43,44,45], and its environmental and health impacts have attracted much attention. Moreover, PAHs emitted from biomass burning can mix with other burning products, such as lignin-derived products (veratraldehyde and vanillic acid) and flavonoid compounds [46]. Photostable compounds (e.g., lignin-derived compounds) in the mixture can inhibit the photodegradation of PAHs and prolong their atmospheric life, enabling them to be transported [47,48,49]. In addition, the enhanced bioactivation of the PAH mixture can cause severe health hazards to humans [50]. Epidemiological studies in many countries (such as China and India) have shown that emissions from biomass burning are likely to increase the prevalence of nasopharyngeal, pulmonary tuberculosis, chronic obstructive pulmonary disease and laryngeal cancer among local residents [51,52,53]. To control the environmental and health side effects from global decarbonization, a comprehensive assessment of PAH emissions from biomass burning is urgently needed.

This review summarizes the concentration and composition of PAHs emitted from biomass burning. The effects of the biomass composition, including volatile matter, nonvolatile components, potassium salts, moisture content and density on PAH formation, are discussed. In addition, the influences of temperature and oxygen supply of burning in stoves on PAH emissions are studied. This review provides a basis for future research on reducing PAH emissions from biomass burning during the global carbon-neutral transition.

## 2. PAH Formation during Biomass Burning

Biomass burning can theoretically be divided into four steps—dehydration, devolatilization, char burning, and ashing [55]. The burning temperature of biomasses ranges from 250 °C to 1200 °C [56]. Under the action of external heating, moisture is volatilized first. As the temperature increases, volatile components in the biomass are volatilized and are expected to be transformed entirely into CO_2_ and H_2_O under high temperatures and aerobic conditions. The remaining char in the biomass burns in the presence of adequate oxygen to form gases (mainly CO and CO_2_) and ashes.

However, many factors affect the complete burning of biomass in the actual burning process; these factors are mainly related to the simultaneous occurrence of theoretical burning steps [57]. For example, the transition from dehydration to devolatilization co-occurs with char burning, which decreases the burning temperature [58]; the concurrent devolatilization and char burning leads to insufficient oxygen supply for both processes [59]. The supplied oxygen reacts preferentially with volatile matter around the biomass fuel and then reacts with char in the biomass, which results in incomplete combustion of both volatile matter and char under high temperatures and insufficient oxygen conditions. These processes are beneficial to the production of PAHs.

In addition, the structural materials (such as lignin) of biomass can produce volatile gases through pyrolysis during char burning decomposition [60,61]. The volatile gases will further react with char to produce gaseous products, including small hydrocarbon radicals (such as ethynyl and 1,3-butadiene radicals) and hydrocarbons (such as propylene and butadiene) [62]. After that, the gaseous product will be converted to LMW PAHs through incomplete combustion during devolatilization. The LWM PAHs will be gradually pyrosynthesized into medium MW (MMW) and high MW (HMW) PAHs through the “zig-zag addition process” [62].

Based on the difference between the theoretical and actual processes of biomass burning, the generation of PAHs is affected mainly by biomass properties and combustion conditions. To comprehensively understand the generation and emission of PAHs in biomass combustion, relevant studies are summarized in the following sections to analyze the emission characteristics of PAHs and identify specific influencing factors.

## 3. PAH Emissions from Biomass Burning

According to burning methods, burning can be mainly divided into stove burning (such as industrial boilers and household stoves) and open burning (such as forest fires, grassland fires and open burning of straws). The characteristics of PAH emissions from various burning methods are different. This study is focused on the 16 PAHs listed by the US Environmental Protection Agency (EPA) as priority air pollutants among the other PAHs. The sixteen PAHs are divided into five groups based on ring number: two-ring PAH (naphthalene), three-ring PAHs (acenaphthylene, acenaphthene, fluorene, phenanthrene and anthracene), four-ring PAHs (fluoranthene, pyrene, benz[*a*]anthracene and chrysene), five-ring PAHs (benzo[*b*]fluoranthene, benzo[*k*]fluoranthene, benzo[*a*]pyrene) and six-ring PAHs (benzo[*g*,*h*,*i*]perylene, indeno[1,2,3-*c*,*d*]pyrene and dibenz[*a*,*h*]anthracene). The 16 species receive much attention due to their prevalence, persistence and potential toxicity to humans [63]. The LMW PAHs include two- and three-ring PAHs, MMW PAHs include four-ring PAHs, and HMW PAHs include five- and six-ring PAHs. It should be noted that PAHs with different MW have different oxidative potentials in human metabolism. PAHs with a relatively high MW positively correlate with high oxidative potential, which leads to severe DNA damage and disturbances in DNA replication, causing higher health risks to humans [64,65].

Table 3 lists several studies that compared particulate matter (PM)-bound PAHs in the periods of non-burning and open burning of crop residues. The PAH emissions from biomass open burning in Hanoi, Vietnam, in spring-summer and autumn-winter show a significant increase when comparing those in the non-burning period (2.60 ± 1.31 ng/m^3^ and 14.1 ± 3.69 ng/m*^3^*, respectively) with those in the open-burning period (3064 ± 2370 ng/m^3^ and 4488 ± 3850 ng/m^3^, respectively) [53]. The concentrations of PAHs in other regions during the open-burning period also show massive increases; concentrations increase approximately 2.80–9.54 times compared to those in the non-burning period [41,66,67], indicating that the open burning of crop residues significantly increases the ambient concentration of PAHs, which can cause severe health effects. Unlike the artificial burning of crop straw, the open burning of biomass in sparsely populated areas is dominated by forest and grassland fires, which increase the local level of PAHs and their load in long-range transport [68,69,70,71]. Wang et al. estimated that the annual emission of PAHs from bushfires/wildfires in Australia ranged from 160 to 1100 Mg/year with a median of 700 Mg/year [72]. The PAHs emitted from wildfires reached approximately 13.6% of the total PAH emissions, and exacerbated global warming could increase the occurrence of wildfires to emit more PAHs [35].

Cooking is the most common situation where biomass is burned in stoves. Table 4 compares the PAH emissions from stove burning of biomass during the cooking period with those during the non-cooking period. The biomass burned in homemade clay-stoves called “Chulha” (no chimney) in Lucknow, India, showed higher PAH concentrations during the cooking period in summer and winter (9110 ± 3570 ng/m^3^ and 15,600 ± 2950 ng/m^3^, respectively) than during the non-cooking period (1120 ± 190 ng/m^3^ and 3530 ± 890 ng/m^3^, respectively), and the PAH emission concentrations were approximately 8.13 and 4.41 times higher during the cooking period in summer and winter, respectively, leading to severe health risks [73]. The biomass burning in brick stoves with a chimney in Laiyang, China, shows a lower increase in the cooking period (696 ± 230 ng/m^3^) than in the non-cooking period (513 ± 225 ng/m^3^) [74]. Moreover, reports show that PAH emissions based on benzo[*a*]pyrene equivalent quantities from biomass burning in domestic stoves for heating and cooking reach approximately 56.7%, which is far higher than emissions from coal (11.7%) [36].

In winter, burning biomass for residential heating was reported as a critical air pollutant emission source [77]. The reports in Europe and North America showed burning biomass for heating had a significant impact on the health of local residents [78,79]. Table 5 compares the particulate matter (PM)-bound PAHs during the non-heating period with the heating period. The PAHs concentration in Zagreb, Croatia in the heating period (41.2 ng/m^3^) was approximately 76.3 times higher than in the non-heating period (0.54 ng/m^3^), which significantly enhanced the health risks of biomass burning to the local residents [80]. In addition, due to the indoor biomass burning for heating, the indoor PAH concentrations were higher during the non-heating (131 ng/m^3^) and heating periods (461 ng/m^3^) than those outdoors (36.0 ng/m^3^ and 106 ng/m^3^, respectively) [81]. In a rural site in Delnice, Croatia, the PAH concentration was 2.23 times higher than the in urban site during the heating period [82]. This difference might be caused by the use of natural gas or oil for residential heating in urban areas, but biomass in rural areas.

Table 6 shows the increase in the PM-bound PAH emissions in the open-burning period compared with those in the non-burning period. Compared with normal periods, the open-burning period displays greater increases in LMW and MMW PAHs than in HMW PAHs, indicating that high levels of LMW and MMW PAHs were formed during biomass burning. In addition, the burning temperature can also lead to massive emissions of LMW and MMW PAHs. Table 7 shows the ignition, peak and burnout temperatures for different biomasses, in which the burning temperatures are approximately 300 °C to 500 °C. Studies have shown that LMW and MMW PAHs are likely to be emitted at low temperatures (<500 °C) [85].

## 4. Factors That Influence PAH Emissions

### 4.1. Types and Compositions of Biomass

Table 8 compares the PAHs emitted from the burning of various types of biomass, which shows that the burning of different types under the same conditions leads to different PAH EFs. For example, the PAH EFs of wheat straw (65.2 mg/kg) burning in brick cook stoves were approximately three times lower than those of corn straw (19.0 mg/kg) [90]. Dung cakes in open burning have a higher total PAH EF value than charcoal (53.8 mg/kg and 27.3 mg/kg, respectively) [91]. Based on the previously described PAH formation during biomass burning, biomass composition, such as volatile and nonvolatile components and moisture can influence the reaction conditions and progress, thus changing the concentration and composition of PAHs emissions. The chemical composition of biomass varies with biomass type [92]. Briefly, the emission characteristics of PAHs are mainly affected by volatile matter, nonvolatile matter, potassium salts, moisture and density of the biomass.

#### 4.1.1. Volatile Matter

Biomass with high volatile contents can generate abundant phenyl radicals during burning, leading to massive PM-bound PAH emissions (Table 9). The PAH EFs of the same biomass burned in the same conditions increase with increasing volatile matter. Based on this feature, the emission of PAHs can be effectively reduced by pretreatments (briquettes or carbonization) that reduce the volatile content of the biomass. For example, Lea-Langton et al. reported that the higher volatile content of firewood (82.6%) than of charcoal (16%) leads to higher PAH emissions (73.4 ng/m^3^ and 7.9 ng/m^3^, respectively) [95]. Moreover, Sun et al. reported that the PAH emissions from burning carbonized maize and wheat were approximately 85% and 88% lower than those from burning unpretreated biomass fuel, respectively [96]. The significant decrease might be due to the briquette and carbonization pyrolyzing and volatilizing more than 80% of the volatile matter in biomass, which decreases the formation of phenyl radicals and PAHs during burning [97,98]. In addition, after discharge into the atmosphere, weather conditions will change the concentration of PAHs [99]. Studies have shown that temperature, ozone, pressure, and relative humidity constituted 43–70% of variability in PAH concentrations [100]. The concentration of PAHs increases with decreasing temperature and decreases with increasing humidity and pressure. The higher ozone concentration can increase the concentration of LMW and MMW PAHs but decrease that of HMW PAHs. Compared with ozone oxidation, LMW and MMW PAHs are more susceptible to photo-oxidation and thermal decomposition [100].

#### 4.1.2. Nonvolatile Matter

In addition to volatile components, nonvolatile components in biomass are essential carbon sources for PAH formation. The pyrolysis of structural materials (such as cellulose and lignin) is the main formation process for PAHs during biomass burning [102,103]. Studies have shown that an increase of 200 mg to 500 mg in cellulose content can cause an increase of 14 μg/g to 24 μg/g in EFs of PAHs during burning [104]. Moreover, the burning of biomass with a high polyunsaturated fatty acid to saturated fatty acid (PUFA/SFA) ratio can lead to high PAH emissions [105,106]. This is because PUFAs have more carbon–carbon double bonds than SFAs, making it easier for them to form free radicals during pyrolysis and, therefore, promoting the generation of PAHs [107].

#### 4.1.3. Potassium Salts

Potassium salts in biomass are considered to play a role in the formation of PAHs during biomass burning. Studies have shown that compared with rice straw containing low potassium (0.57 g/kg), wheat straw with high potassium content (31.3 g/kg) increases PAH emissions from burning by approximately three-fold (0.362 mg/g OC and 1.2 mg/g OC, respectively) [108]. During burning, inorganic potassium salts act as catalysts during the pyrolysis of lignin, which accelerates the pyrolysis of lignin into phenyl radicals and then forms PAHs through pyrosynthesis [108,109]. This finding indicates that biomass containing pyrolysis-promoting components tends to discharge more PAHs and highly toxic PAHs during burning. Therefore, the improvement and optimization of biomass is crucial to controlling PAH pollution caused by biomass burning. 

To reduce the pyrolysis-promoting components in biomass, one possible solution is to wash the biomass with deionized water. Deionized water has been reported to extract minerals such as potassium, terpenoids and phenolic compounds from biomass, thus reducing the emission of PAHs during burning by approximately 60% [110]. A report showed that the PAH emission (1570 μg/kg_dw_) from the burning of raw fir was much higher than that (560 μg/kg_dw_) from the burning of fir washed with deionized water. In addition, washing with deionized water can change the composition of the discharged PAHs, which emit fewer HMW PAHs (five-ring) but higher LMW and MMW PAHs. For instance, after washing with deionized water, the proportion of LWM and MMW PAHs to the total PAHs increased from 88% (raw fir) to 99% (washed fir), causing toxic potency of emitted PAHs decreased by approximately 96% [110]. The results indicate that inorganic potassium salts can catalyze the pyrolysis, and washing the biomass with deionized water before burning can significantly lower the associated health risks to humans.

#### 4.1.4. Moisture

Table 10 summarizes the changes in the concentration and composition of PAHs emitted from burning caused by changing the moisture content of biomass. Most of the results show an increase in the EFs of PAHs with increasing biomass moisture. As previously described, dehydration at the beginning of biomass burning leads to an oxygen-deficient atmosphere [111]. Guerrero F. et al. proved that an increase in biomass moisture content from 0% to 25% results in more CO being formed (maximum peaks of 2007 ppm and 3742 ppm, respectively) than CO_2_ [112]. These changes in burning conditions can cause incomplete oxidation of biomass, leading to the massive generation of PAHs during subsequent devolatilization and char burning. However, the results of Korenaga et al. (Table 10) showed that as the biomass moisture increased, the EFs of PAHs first decreased and then increased. This trend might imply that the dehydration step in the burning process is skipped during the burning of biomass containing no moisture, leading to an insufficient oxygen supply in a closed draft chamber. The insufficient oxygen supply leads to incomplete combustion of biomass, thus increasing the generation and emission of PAHs [93,113,114]. Moreover, the study showed that a lower biomass moisture content could increase the temperature in burning (maximum peaks of 537 °C and 236 °C for 0% and 25% moisture contents, respectively), which caused higher PAH formation [112,115].

#### 4.1.5. Density of Biomass

In addition to the biomass components mentioned above, the density of biomass also affects the burning efficiency and PAH emissions [54]. At present, the pelletizing and briquetting of biomass has become an effective method for increasing the density of biomass [118]. The processes of pelletization and briquetting of biomass fuel are similar and include crushing, drying and pelletizing [119]. These processes can increase the biomass density, leading to a reduction in the burning rates to inhibit the incomplete combustion of biomass [120]. Moreover, the pelletizing and briquetting of biomass can decrease the contents of moisture and volatile matter, which further reduces PAH emissions [121]. The use of pelletized biomass has been reported to reduce total PAH emissions by approximately 89% (in the range of 69% to 94%) and to reduce emissions of toxic benzo[*a*]pyrene by approximately 89% (in the range of 60% to 94%) [122]. These measures can effectively decrease the health risks of biomass burning to humans.

### 4.2. Burning Conditions

Table 11 summarizes the EFs of PAHs emitted from biomass burning with different types of stoves. The burner is an exogenous factor that affects burning conditions (such as temperature and oxygen supply) and PAH emissions during biomass burning. Traditional domestic heating and cooking stoves (such as fireplaces, woodstoves, and brick stoves) normally have low burning efficiency and emit high PAHs during burning [123]. For example, the heating stove called Heated Kang is widely used in China. The structure of Heated Kang is a burning chamber (Kang) connected to a chimney by a tube, and there is no secondary air system to supply oxygen (or heated oxygen) to the burning chamber during burning [108]. Insufficient ventilation usually reduces the burning efficiency of biomass and increases the emission of PAHs and their adverse effects on human health [124,125,126]. Compared with traditional domestic stoves, improved stoves (such as pellet stoves and gasifier stoves) can decrease incomplete combustion due to the advanced ventilation system and stove temperature control system [127].

#### 4.2.1. Burning Temperature

The burning temperature of the stove strongly affects the composition of the PAHs that form. For instance, Table 12 summarizes the increased rate of PAHs emissions by the same stove with the same oxygen supply at different temperatures relative to the PAHs emitted at 200 °C [131]. The results from laboratory simulations showed that the LMW and MMW PAHs begin to be emitted in great quantities during biomass burning with temperatures higher than or equal to 400 °C, and HMW PAHs start to form mainly at higher temperatures (≥ 500 °C) [104,132]. In addition, the emission of PAHs increases with increasing temperature in a specific range, and a higher temperature helps to better synthesize PAHs from fragments that are pyrolyzed from biomass fuel during burning [81].

#### 4.2.2. Oxygen Supply

At a constant temperature, adjusting the oxygen supply can increase the air–fuel ratio in the stove to suppress incomplete combustion and reduce PAH emissions. Increasing the airflow is a commonly used way of improving the oxygen supply. Vicente et al. compared the PAHs emitted from biomass burning in fireplaces and woodstoves by burning the same biomass with the same amount of biomass [133]. In contrast to the fireplace, the woodstove added an extra ventilation pipe to draw fresh air, thereby increasing the air–fuel ratio to reduce incomplete combustion and PAH emissions [124,125]. As a result, burning in the fireplace led to a higher PAH concentration (92.0 ng/m^3^) than burning in the woodstove (8.82 ng/m^3^) [126]. The results from another study also confirmed that biomass burning in a fireplace resulted in higher PAH emissions (80.6 ng/m^3^) than burning in a woodstove (69.1 ng/m^3^) [134]. These findings highlight the positive role of optimizing the oxygen supply efficiency of stoves in reducing both PAH emissions from biomass burning and the associated health risks to humans. However, a rapid increase in the airflow in stoves during burning in actual use may decrease the air–fuel ratio and increase PAH emissions. Wei et al. compared the PAHs emitted from biomass burning in two similar stoves with different stove ages (1 year versus 15 years) [135]. Compared with the 1-year-old stove, the 15-year-old stove had a worse flue block, which reduced the oxygen supply and burning temperature. However, the measured PAH EF of the 1-year-old stove (330 mg/kg) was significantly higher than that of the 15-year-old stove (190 mg/kg). This result suggests that an abundant oxygen supply can accelerate biomass burning, but a surge may lead to rapid burning (i.e., incomplete combustion). Nevertheless, biomass can be converted into gaseous organic molecules such as methane and acetylene by heating it in the absence of oxygen prior to combustion [136,137]. This treatment can effectively improve the combustion efficiency of gaseous organic molecules compared to burning biomass directly, thereby reducing PAHs emissions and producing valuable materials such as graphene [138]. In addition, using supercritical water as an oxidant during biomass gasification can generate hydrogen and carbon monoxide, and reduce the emission of PAHs during combustion [139,140,141]. Therefore, the dual control of the oxygen supply and rate of the burner is considered to reduce the emission of PAHs from biomass burning and the related health risks.

#### 4.2.3. Stove Designs

Based on the effect of temperature and oxygen supply during biomass burning on PAHs emissions, the designs of stoves become a critical factor. An improper stove design can lead to low thermal performance and high emission of air pollutants [142]. A report shows that the space size and the fuel load of the stove can affect the PAHs emission [143]. Less free space in the stove and high loaded fuel cause the temperature in the stove to rise rapidly during biomass burning (flue gas temperature was 143 °C and 187 °C for partial load and full load, respectively), which produces more HMW PAH than burning at low temperatures. Moreover, a substandard airflow path can cause insufficient mixing of oxygen with fuel. Szramowiat-Sala K. et al. compares PAH EFs from burning the same biomass in the same stove with different airflow paths [144]. The EFs of PAHs from burning biomass in the stove with the better airflow path (air inlet located below the door, above the doors and at the rear wall of the stove; EFs = 2.20 mg/kg) was significantly lower than those in the stove with a worse airflow path (air inlets located only below the doors and above the doors; EFs = 10.0 g/kg).

## 5. Conclusions

This review summarizes the related research on PAH emissions from biomass combustion and discusses the effects of biomass composition and combustion conditions on PAH emissions based on the formation mechanism and emission characteristics of PAHs. 

Biomass with high volatile matter content can increase the formation of phenyl radicals that increase the PAH emissions. The high cellulose content and PUFA/SFA ratio of biomass increase the forms of free radicals that enhance PAH EFs. Potassium salts in biomass can act as catalysts for PAH formation. Burning of biomass with a high moisture content causes oxygen deficiency during combustion, which increases PAH formation and leads to a higher health risk. Increasing the biomass density can reduce the biomass burning rates and decrease incomplete combustion leading to low PAH emissions. High burning temperatures can increase the formation of HMW PAHs, causing severe health effects to humans, and optimizing the oxygen supply efficiency of stoves can significantly decrease PAH formation during burning.

To control the incomplete combustion of biomass and the emissions of PAHs, we recommend washing the biomass with deionized water and pelletizing, briquetting or carbonizing the biomass after drying the washed biomass. Furthermore, the domestic stove burning temperature should be controlled at a relatively low degree (<500 °C), and the oxygen supply should be regulated without a rapid increase in the airflow.

## Figures and Tables

**Table 1:** Top three industries in PAHs emissions in different countries and around the world.

Region	First PAH Emissions ^1^		Second PAH Emissions ^1^		Third PAH Emissions ^1^	
	Industry	Amount (Gg)	Industry	Amount (Gg)	Industry	Amount (Gg)
World	IBB ^2^	293	Motor Vehicle	64.5	Wildfire	56.4
China	IBB	57.7	Coke burning	13.8	Motor Vehicle	13.4
India	IBB	55.8	Motor Vehicle	5.03	BOB ^3^	2.35
Brazil	Wildfire	17.4	IBB	5.13	Motor Vehicle	4.35
Indonesia	IBB	15.4	Motor Vehicle	2.89	Wildfire	0.43
Russia	Motor Vehicle	2.83	Wildfire	1.87	Industry	1.11
Angola	Wildfire	3.49	IBB	0.86	Motor Vehicle	0.14
The United States	IBB	4.96	Motor Vehicle	1.50	Wildfire	1.02

**^1^** Shen et al. [35]; ^2^ IBB: Indoor biomass burning; ^3^ BOB: Biomass open burning.

**Table 2:** Comparison of the EFs of PAHs emission from traditional fuels and biomass fuels.

Traditional Fuel		Biomass Fuel		Reference
Fuel Type	EFs (mg/kg)	Fuel Type	EFs (mg/kg)	
Anthracite	2.1	Mixed wood	60.6	[40]
Mixed coal	119.1	Mixed wood	38.9	[43]
Mixed coal	15.5	Crop residue pellets	43.9	[44]
Anthracite and bituminous	123.1	Crop residue and wood	191.1	[54]

**Table 3:** Comparison of the concentration of PAHs in non-burning period with open burning period.

PAHs Number	Cities	Non-Burning Period (ng/m^3^)	Open Burning Period (ng/m^3^)	Reference
16	Taichung, China	11.0 ± 1.51	30.8 ± 3.93	[41]
9	Hanoi, Vietnam	14.4 ± 3.69	4488 ± 3850	[53]
9	Hanoi, Vietnam	2.60 ± 1.31	3064 ± 2370	[53]
16	Bangkok, Thailand	32.4 ± 17.1	108 ± 25.7	[66]
14	Klong Luang, Thailand	43.4 ± 20.0	414 ± 24.0	[67]

**Table 4:** Comparison of the concentration of PAHs in non-cooking with cooking.

PAHs Number	Cities	Non-Cooking (ng/m^3^)	Cooking (ng/m^3^)	Reference
7	Lucknow, India	1120 ±190	9110 ± 3570	[73]
7	Lucknow, India	3530 ± 890	15600 ± 2950	[73]
15	Laiyang, China	513 ± 225	696 ± 230	[74]
16	Nanyang, China	210 ± 23.4	443 ± 59.7	[75]
15	Zhuanghu, China	1530 ± 244	2660 ± 1120	[76]

**Table 5:** Comparison of PAHs concentration in the non-heating period with heating period.

PAHs Number	Cities	Non-Heating Period (ng/m^3^)	Heating Period (ng/m^3^)	Reference
10	Zagreb, Croatian	0.54	41.2	[80]
15	Warsaw, Poland (Outdoor)	36.0	106	[81]
15	Warsaw, Poland (Indoor)	131	461	[81]
10	Delnice, Croatia (Rural)	0.80	48.5	[82]
10	Delnice, Croatia (Urban)	0.40	21.7	[82]
9	Rome, Italy	1.28	10.6	[83]
9	Augsburg, Germany	0.44	13.0	[84]

**Table 6:** Composition of the PAHs in different studies in normal period and biomass burning period.

City	4-Ring PAHs (ng/m^3^)	5-Ring PAHs (ng/m^3^)	6-Ring PAHs (ng/m^3^)	Total PAHs (ng/m^3^)	Reference
Tainan, China	6.64	0.56	1.12	2.83	[49]
Hanoi, Vietnam	401	219	86.0	311	[53]
Baoding, China	3.89	3.58	3.27	3.59	[86]

**Table 7:** The ignition, peak and burnout temperature of biomass.

Biomass	Ignition (°C)	Peak (°C)	Burnout (°C)	Reference
Oat straw	260	300	512	[87]
Wheat straw	227	281	436	[88]
Wheat husk	242	299	490	[88]
cotton stalks	261	294	480	[89]

**Table 8:** Emission factors and composition of PAHs from the burning of different biomass in the same conditions.

	3-Ring	4-Ring	5-Ring	6-Ring	Total PAHs (mg/kg)	Stove	Reference
Rice Straw	49%	38%	8%	4%	42.5	Brick cooking stove	[90]
Wheat Straw	63%	28%	6%	3%	65.2		
Corn Straw	60%	30%	7%	3%	19.0		
Dung Cakes	30%	44%	16%	11%	53.8	Open burning	[91]
Charcoal	21%	47%	19%	13%	27.3		
Crop residue	66%	26%	6%	3%	30.0	Brick cooking stove	[93]
Fuel wood	59%	33%	5%	2%	6.76		
Brushwood	38%	42%	12%	7%	47.1		
Ceanothus	37%	34%	23%	6%	6.47	Laboratory burning stove	[94]
California sage	5%	47%	39%	8%	11.7		
Coastal sage	6%	49%	30%	15%	11.3		

**Table 9:** The emission of PAHs under the burning of same biomass contains different volatile matter in the same stove during the laboratory-simulated stove burning.

Biomass	Volatile Matter (wt%)	PAHs (μg/g)	Reference
Maize straw (Raw)	76.00	7.70	[96]
Maize straw (Carbonization)	25.01	1.10	
wheat straw (Raw)	67.36	18.8	
wheat straw (Carbonization)	16.95	1.60	
Wood branch (Raw)	82.96	8.70	
Wood branch (Carbonization)	44.94	2.90	
Wheat straw (Raw)	73.27	213	[101]
Wheat straw (Carbonization)	29.99	13.1	
Rice Straw (Raw)	71.53	189	
Rice Straw (Carbonization)	25.67	18.2	
Maize Straw (Raw)	74.22	203	
Maize Straw (Carbonization)	28.26	4.03	
Sawdust (Raw)	77.47	241	
Sawdust (Carbonization)	38.44	4.73	

**Table 10:** The EF and composition of PAHs at different biomass moisture content.

Moisture (%)	Total PAHs (mg/kg)	Reference
0	3.19 × 10^−3^	[112]
25	5.57 × 10^−3^	
0	33.87	[115]
5	4.22	
10	2.42	
15	1.75	
20	4.06	
25	3.43	
30	5.42	
5	3.02	[116]
10	8.14	
20	17.1	
15	3.20	[117]
25	24.3	

**Table 11:** Emission factors of PAHs of the same biomass burning in different stoves.

Stove Type	PAHs EFs (mg/kg)	Reference
Top-feed pellet stove	0.04	[128]
Wood stove	0.96	
Gasifier wood stove	31.2	[129]
Guizhou brick stove	132	
Sichuan brick stove	262	
Metal stove	5.50	[130]
Grihalaxmi stove	3.80	
Traditional stove	3.10	

**Table 12:** Relative changes in PAH composition from rice and bean burning at different temperatures.

PAHs	R_300/200_^1,2^	R_400/200_^2^	R_500/200_^2^	R_600/200_^2^	R_700/200_^2^
Rice					
3-ring	1.48	1.50	1.74	2.52	2.63
4-ring	1.68	2.43	3.72	6.26	7.35
5-ring	1.81	1.95	2.54	3.04	3.15
6-ring	2.33	1.56	2.24	2.11	3.35
Bean					
3-ring	1.06	1.28	3.54	5.17	16.1
4-ring	0.87	1.19	2.04	4.10	9.16
5-ring	0.75	0.99	1.83	2.93	19.7
6-ring	1.01	1.04	1.45	2.46	24.5

^1^ Represents the result of PAHs EF values of biomass burning in 300 °C divided with PAHs EF values of biomass burning in 200 °C; ^2^ Lu et al. [131].

## Data Availability

Data sharing not applicable.
